# Computerized attention training for visually impaired older adults with dementia: a case study

**DOI:** 10.1590/1980-57642020dn14-040015

**Published:** 2020-12

**Authors:** Michael Chih Chien Kuo, Tsz Yang Fong, Cheuk Wing Fung, Chi To Pang, Lok Man So, Ka Ki Tse, Armstrong Tat San Chiu, King Yeung

**Affiliations:** 1 School of Medical and Health Sciences, Tung Wah College – Hong Kong SAR, China.; 2 The Hong Kong Society for the Blind – Hong Kong SAR, China.

**Keywords:** computer user training, attention, visual impairment, dementia, capacitação de usuário de computador, atenção, transtornos da visão, demência

## Abstract

Dementia causes disorders in multiple higher cortical functions. Visual impairment could further impact cognition in those with dementia. This study reports the results of a computerized attention training program in a patient with dementia and visual impairment. The case involves a 98-year-old woman with bilateral maculopathy and moderate dementia. The program consisted of pre- and post-assessments and training sessions. Assessments included the Cantonese version of the Mini-Mental State Examination, the digit span forward test, the Chinese version of the Verbal Learning Test (CVVLT), and the Test of Attentional Performance (TAP). Training sessions were conducted once to twice a week for a total of 8 45-minute sessions. The participant showed a decrease in the CVVLT score and improvements in TAP parameters. The results indicated that, in visually impaired older adults with dementia, attention and processing speed (measured by a sensitive test such as TAP) could potentially be improved with appropriate computerized training.

## RESUMO.

A demência causa distúrbios em várias funções corticais superiores. O comprometimento da visão pode impactar ainda mais a cognição em pessoas com demência. Este estudo relata os resultados de um programa de treinamento computadorizado de atenção em um paciente com demência e deficiência visual, uma mulher de 98 anos com maculopatia bilateral e demência moderada. O programa consistia em avaliações e pré- e pós-sessões de treinamento. As avaliações incluíram o Mini-Exame do Estado Mental em Cantonês, o teste de extensão de dígitos, a versão chinesa do Teste de Aprendizagem Verbal (CVVLT) e o Teste de Desempenho de Atenção (TAP). As sessões de treinamento foram realizadas uma a duas vezes por semana, com um total de 8 sessões de 45 minutos. A paciente apresentou diminuição no escore CVVLT e melhora nos parâmetros TAP. Os resultados indicaram que em idosos com deficiência visual com demência, a atenção e a velocidade de processamento (medida por um teste sensível como o TAP) poderiam ser melhoradas com a realização de treinamento computadorizado adequado.

## INTRODUCTION

Dementia is a chronic and progressive brain disease that causes disorders in multiple higher cortical functions, including attention and memory. Estimates indicate that 46.8 million people worldwide are living with dementia. This number is expected to double every 20 years.^1^ Worsening vision, a common condition among older individuals, is associated with declining cognitive function and could produce an additional impact on people with dementia.^2^ ^,^ ^3^ Current medical and conventional treatments are unable to cure or reverse the decline in cognitive function in dementia.^4^ In recent years, computerized cognitive training has become more popular, as it is a safe, relatively inexpensive, and beneficial way to maintain cognitive function in older adults with mild to moderate dementia.^5^ ^–^ ^8^

Attention and memory, though distinct, are interrelated cognitive processes.^9^ Attention is required for memory encoding and storage, including acoustic information.^10^ While many studies have reported on the use of memory training in people with cognitive disorders,^6^ ^,^ ^11^ very few have investigated attention training and how it can affect attention and memory functions in older people with dementia who also have visual impairment. This study describes the results of a computerized attention training program in a visually impaired patient with moderate dementia. To avoid potential difficulties related to the participant's visual impairment, only attention training tasks that provided auditory cues were selected. In addition to typical neurocognitive assessments (e.g., Mini-Mental State Examination, digit span test, and immediate and delayed recall tests), a subtest of the Test of Attentional Performance (TAP)^12^ that assessed divided attention with auditory stimuli was completed by the patient.

## METHOD

The case involved a 98-year-old woman, who had been staying in a local care home for older people since 2014. She received 9 years of education and was diagnosed with right and left maculopathy and moderate dementia. She had no other neurological conditions. She was in good general health, had intact hearing, and was able to follow simple instructions consistently to complete the assessments and the training. This study was approved by the ethics committee of the academic institution.

The program consisted of ten sessions — one initial (pre-assessment) session, eight training sessions, and one final (post-assessment) session. Neuropsychological assessments included the Cantonese version of the Mini-Mental State Examination (CMMSE),^13^ the digit span forward test,^14^ the Chinese version of the Verbal Learning Test (CVVLT),^15^ and the divided attention subtest of the TAP.^12^ CMMSE (maximum score of 30) was used for screening the overall cognitive function. Digit span forward test (maximum score of 14) was used for selective attention. CVVLT consisted of 9 two-Chinese character nouns with 4 learning trials (maximum score of 36) and 1 10-minute delayed recall trial (maximum score of 9). TAP required the use of a response pad. In the test, high- and low-pitched ‘bi’ sounds were played alternatively, and the participant was instructed to press the button on the pad when she identified these same pitches (either high or low) consecutively. The patient was evaluated based on the rate of response accuracy (i.e., number of correct responses, number of wrong responses, and number of omissions) and mean reaction time (mean time to react to correct stimuli). A higher score or rate of response accuracy and lower mean reaction time would indicate better performance.

Training sessions were conducted once to twice a week for a total of 8 sessions. Each session lasted approximately 45 minutes and included 30 minutes of actual training tasks. Selective and focused attention modules of the CogniPlus (Schuhfried GmbH, Mödling, Austria) were used alternatively from one training session to another. Vision is not explicitly required to complete these modules; thus, individuals with visual impairment are not prevented from participating in any way. Before the training tasks, rapport was established, and instructions on the task-taking process were provided to the participant. Reinforcements/cues were given throughout the training to promote participation. The completion of the task in each training session was followed by a summary of what the participant had accomplished. The CogniPlus program remembers and adjusts the levels of challenge automatically according to the individual's performance. The patient started at the easiest difficulty levels on both tasks, and by the end of eight training sessions, she had progressed to medium-to-hard difficulty levels.

## RESULTS

**Figure 1:**
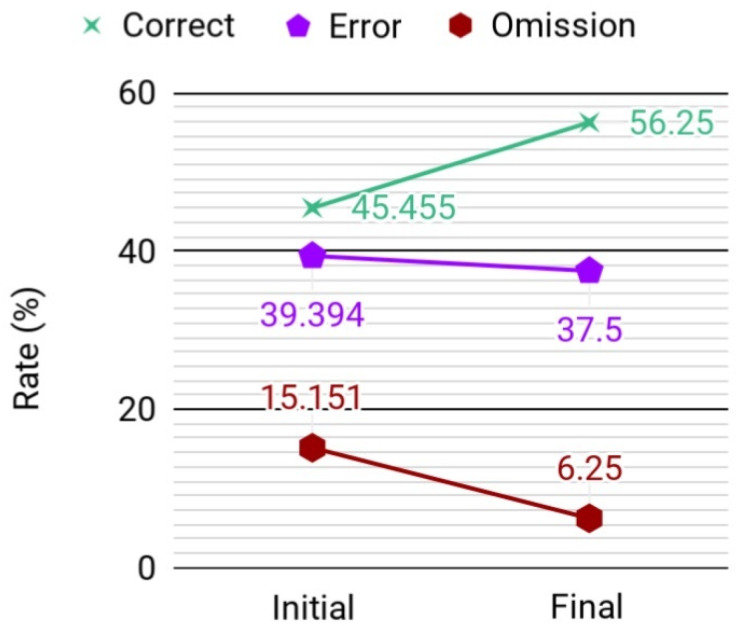
Changes in correct, error, and omission rates.

**Figure 2:**
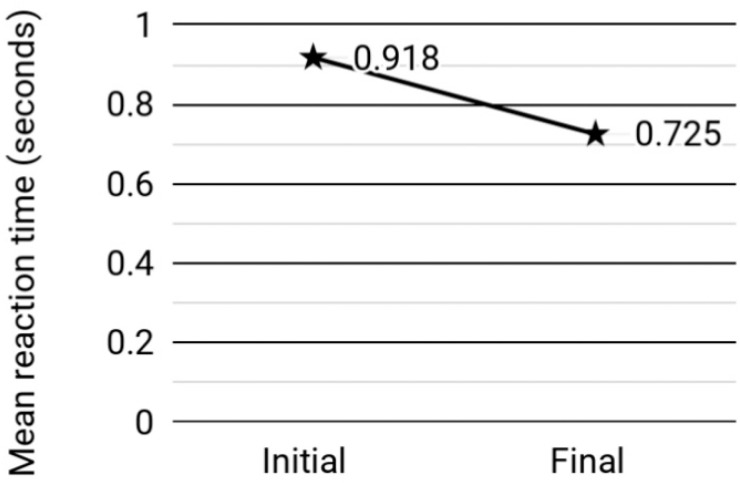
Change in reaction time.

The participant's CMMSE score remained at 17 (out of 30) between pre- and post-assessments. The score for the digit span forward test continued the same as well (11 out of 14). The total number of character nouns recalled over the 4 immediate recall trials in CVVLT decreased from 15 to 9, while the delayed recall score remained the same (0 out of 9). In TAP (see Figures 1 and 2), the number of correct responses changed from 15 out of 33 in the pre-assessment to 18 out of 32 in the post-assessment (10.8% increase). The number of errors changed from 13 out of 33 to 12 out of 32 (4.7% decrease). The number of omissions changed from 5 out of 33 to 2 out of 32 (58.7% decrease). The mean reaction time changed from 0.918 to 0.725 seconds (21% decrease).

## DISCUSSION

This study reports the results of the effect of a computerized attention training program on a woman with moderate dementia and visual impairment. The participant showed no changes in CMMSE and digit span scores, decrease in CVVLT score, and some improvements in TAP parameters.

Most studies on computer-based training programs for people with dementia have focused on the memory domain and relied on their intact vision.^6^ ^,^ ^16^ We found no specific studies of attention training on moderate dementia with visual impairment. However, a recent report investigating the effect of attention training on improving selective, focused, and divided attention for older adults with mild cognitive impairment (MCI) revealed significantly improved performance in attention after the training.^17^ MCI comprises a clinical group with a significantly better cognitive profile than people with dementia and, thus, more potential for improvement. Nevertheless, the results from this study indicated that, in visually impaired older adults with dementia, attention and auditory-motor processing speed (measured by a sensitive test such as TAP) could potentially be improved with appropriate computerized training. This investigation was limited by its single-case design, and improvements were not identified in global or cognitive domains other than those tested by TAP. In addition, performance in activities of daily living and behavioral symptoms were not assessed. Therefore, how attention training can influence these aspects in visually impaired dementia patients is unknown. Future larger-scale studies on visually impaired dementia patients could consider using similar training modules and design. Other TAP subtests that assess the performance of auditory tasks could also be considered as outcome measures.

## References

[1] Alzheimer's Disease International (2015). World Alzheimer Report 2015: The Global Impact of Dementia.

[2] Zheng DD, Swenor BK, Christ SL, West SK, Lam BL, Lee DJ (2018). Longitudinal associations between visual impairment and cognitive functioning: the Salisbury Eye Evaluation Study. JAMA Ophthalmol.

[3] Maharani A, Dawes P, Nazroo J, Tampubolon G, Pendleton N, Sense-Cog WP1 group (2018). Visual and hearing impairments are associated with cognitive decline in older people. Age Ageing.

[4] Overshott R, Burns A (2005). Treatment of dementia. J Neurol Neurosurg Psychiatry.

[5] Liang J, Xu Y, Lin L, Jia R-X, Zhang H-B, Hang L (2018). Comparison of multiple interventions for older adults with Alzheimer disease or mild cognitive impairment: a PRISMA-compliant network meta-analysis. Medicine (Baltimore).

[6] Klimova B, Maresova P (2017). Computer-based training programs for older people with mild cognitive impairment and/or dementia. Front Hum Neurosci.

[7] Hill NT, Mowszowski L, Naismith SL, Chadwick VL, Valenzuela M, Lampit A (2017). Computerized cognitive training in older adults with mild cognitive impairment or dementia: a systematic review and meta-analysis. Am J Psychiatry.

[8] Tárraga L, Boada M, Modinos G, Espinosa A, Diego S, Morera A (2006). A randomised pilot study to assess the efficacy of an interactive, multimedia tool of cognitive stimulation in Alzheimer's disease. J Neurol Neurosurg Psychiatry.

[9] Castel AD, Balota DA, McCabe DP (2009). Memory efficiency and the strategic control of attention at encoding: Impairments of value-directed remembering in Alzheimer's disease. Neuropsychology.

[10] Robertson IH, Ward T, Ridgeway V, Nimmo-Smith I (1996). The structure of normal human attention: The Test of Everyday Attention. J Int Neuropsychol Soc.

[11] Yang HL, Chan PT, Chang PC, Chiu HL, Hsiao STS, Chu H (2018). Memory-focused interventions for people with cognitive disorders: a systematic review and meta-analysis of randomized controlled studies. Int J Nurs Stud.

[12] Zimmermann P, Fimm B, Leclercq M, Zimmermann P (2004). A test battery for attentional performance. Applied neuropsychology of attention: theory, diagnosis and rehabilitation.

[13] Chiu HFK, Lee HC, Chung WS, Kwong PK (1994). Reliability and validity of the Cantonese version of Mini-Mental State Examination - A preliminary study. J Hong Kong Coll Psychiatry.

[14] Wechsler D (1997). WAIS-III: administration and scoring manual: Wechsler Adult Intelligence Scale.

[15] Chang CC, Kramer JH, Lin KN, Chang WN, Wang YL, Huang CW (2010). Validating the Chinese version of the verbal learning test for screening Alzheimer's disease. J Int Neuropsychol Soc.

[16] Mueller KD (2016). A review of computer-based cognitive training for individuals with mild cognitive impairment and Alzheimer's disease. Perspect ASHA Spec Interest Groups.

[17] Yang HL, Chu H, Miao NF, Chang PC, Tseng P, Chen R (2019). The construction and evaluation of executive attention training to improve selective attention, focused attention, and divided attention for older adults with mild cognitive impairment: a randomized controlled trial. Am J Geriatr Psychiatry.

